# The Role of Rho GTPases in VEGF Signaling in Cancer Cells

**DOI:** 10.1155/2020/2097214

**Published:** 2020-04-16

**Authors:** Nada El Baba, Mohammad Farran, Elie Abi Khalil, Leila Jaafar, Isabelle Fakhoury, Mirvat El-Sibai

**Affiliations:** Department of Natural Sciences, School of Arts and Sciences, Lebanese American University, Beirut, Lebanon

## Abstract

Vascular endothelial growth factors (VEGFs) consist of five molecules (VEGFA through D as well as placental growth factor) which are crucial for regulating key cellular and tissue functions. The role of VEGF and its intracellular signaling and downstream molecular pathways have been thoroughly studied. Activation of VEGF signal transduction can be initiated by the molecules' binding to two classes of transmembrane receptors: (1) the VEGF tyrosine kinase receptors (VEGF receptors 1 through 3) and (2) the neuropilins (NRP1 and 2). The involvement of Rho GTPases in modulating VEGFA signaling in both cancer cells and endothelial cells has also been well established. Additionally, different isoforms of Rho GTPases, namely, RhoA, RhoC, and RhoG, have been shown to regulate VEGF expression as well as blood vessel formation. This review article will explore how Rho GTPases modulate VEGF signaling and the consequences of such interaction on cancer progression.

## 1. Introduction

Vascular endothelial growth factors (VEGFs) consist of a family of five secreted glycoproteins (VEGFA through D and placental growth factor) [1]. These members form complexes with their corresponding VEGF receptors (VEGFR1 through 3) which then dimerize leading to the activation of their cytoplasmic tyrosine kinase [2]. This series of events regulates endothelial cells as well as angiogenesis, which is the branching of preexisting blood vessels to form new ones. Angiogenesis is a process that is indispensable for embryonic development, growth, regeneration, and wound healing [3, 4]. Additionally, angiogenesis has been associated with abnormal functions and pathologies including arthritis, muscular dystrophy, diabetes, and in context of this review, tumorigenesis [5, 6]. In cancers, the angiogenic signals initiate the branching of endothelial cells (ECs) from preexisting vessels and the formation of new capillaries which will supply the tumors with the required nutrients [4]. Literature has shown that the binding of VEGFs to their corresponding receptors is the main angiogenic stimulus which triggers the formation of new blood vessels [3, 7]. Other growth factors contribute to the proliferation and migration of ECs by activating the phosphatidylinositol 3 kinase (PI3K) pathway as well as the mitogen-activated protein kinase (MAPK) pathway [8–10]. In parallel, the regulation of tumors by the action of the Rho family of GTPases on VEGF signaling has also been demonstrated [11–13].

The Rho family of GTPases consists of 20 members of small GTP-binding proteins with molecular sizes ranging between 20 and 40 KDa [14]. The most well-characterized members are RhoA, RhoC, Rac1, and Cdc42 [15–18]. Rho GTPases regulate several biological processes mainly by remodeling actin and the cytoskeleton [19–21]. Specifically, RhoA, RhoC, Rac1, and Cdc42 can regulate endothelial cell proliferation, polarization, cell-cell adhesion, and migration, as well as vascular permeability during angiogenesis [11, 13, 22–27].

In this review, we will explore the relationships between VEGFs, their receptors, and the Rho GTPases, highlighting the involvement of RhoA, RhoC, and RhoG in VEGF signaling and the formation of new blood vessels in cancer. We will also explore how the crosstalk between VEGF and Rho-related pathways contributes to tumorigenesis and invasion.

## 2. Angiogenesis

Angiogenesis is a complex and well-regulated biological phenomenon which involves branching and remodeling [28]. It is important to distinguish between angiogenesis and vasculogenesis which is a process that takes place during embryonic development and leads to the initial formation of blood vessels from ancestral endothelial cells (ECs) [29]. The formation of new blood vessels and capillaries from preexisting ones is indispensable for many normal physiological functions including wound healing and the menstrual cycle and is commonly deregulated in cancer to supply tumors with sufficient oxygen and nutrients to ensure their survival and growth [28, 29]. Despite the advancements in surgeries and the development of different therapies, angiogenesis remains a major challenge and is associated with tumor aggressiveness and overall higher patient mortality rate. Tumors initiate angiogenesis by releasing VEGF from tumor cells which are found in microenvironments with low oxygen and high interstitial fluid pressure [30]. This process is coordinated by four steps: (1) the activation of ECs by the hypoxia-inducible factor (HIF) which is produced in response to hypoxia or the drop of oxygen levels [28]; (2) the breakdown of the basement membrane by proteases, including matrix metalloproteases (MMPs), catheprins, and plasminogen activators (PAs). This serves as a preparatory step for the formation of the endothelial tubing. [28]; (3) the initiation of the endothelial tube formation in response to the increase in the production of several growth factors following the breakdown of the basement membrane. ECs thus begin to migrate and multiply on site in response to growth factors such as VEGF, basic fibroblast growth factor (bFGF), and platelet-derived growth factor (PDGF) [28, 31]; and (4) the maturation of the newly formed vessels including the formation of the vascular basement membrane and the recruitment of mesenchymal cells, pericytes, and smooth muscle cells to the walls of the newly formed tubes. This step confers the polarity and stability of the capillaries [28].

## 3. VEGF as an Angiogenic Modulator

VEGF was initially characterized as a mitogen involved in physiological angiogenesis, namely, vascular angiogenesis and lymphangiogenesis as well as pathological angiogenesis and vascular permeability in endothelial cells (ECs) [32, 33]. It is well established that these processes are performed by VEGFA, which is still often referred to as VEGF. VEGFA remains the most thoroughly studied factor of the VEGF family of growth factors which is comprised of VEGFA, VEGFB, VEGFC, VEGFD, and the placental growth factor (PIGF). Additionally, these members can have different variants generated by alternative splicing. VEGFA variants for instance include VEGF121, VEGF145, VEGF148, VEGF165, VEGF183, VEGF189, and VEGF206. These factors and variants are involved in a diverse array of biological functions and are unique in their expression patterns and receptor specificity [34]. In tumors, VEGF is produced by hypoxic tumor cells, ECs, and infiltrating myeloid cells which are known as the tumor-associated macrophages (TAMs) [35]. By supplying oxygen and nutrients to the tumors, it is well established that VEGFA enhances tumor progression [36, 37]. Specifically, cancer cell migration, invasion, and angiogenesis can also be linked to the increase in the expression levels of VEGF [38]. This has thus provided the basis for the development of therapies which target angiogenesis in cancer cells by downregulating or inhibiting VEGF and/or interfering with the corresponding (VEGFRs) receptors [1, 39].

## 4. VEGFRs and NRPs

VEGFs induce intracellular signal transduction upon binding to two types of transmembrane receptors: the VEGF receptors (VEGFRs), which are tyrosine kinase receptors (VEGF receptors 1, 2, and 3), or the neuropilins (NRPs) (NRP1 and NRP2) [40]. VEGFRs are type 1 transmembrane proteins that comprise seven extracellular immunoglobulin-like domains, a transmembrane domain, and a cytoplasmic region [41]. These receptors were initially thought to be exclusively expressed in endothelial cells, but their expression has also been reported in many tumor types. Binding of the VEGF ligand to the receptor is specific. For example, VEGFR2 (also named KDR or Flk-1) which is preferentially expressed in vascular endothelial cells is responsible for the transduction of angiogenic signals of VEGFA [41]. Upon binding, the kinase activity of VEGFR2 autophosphorylates the tyrosine residues present in the intracellular domain which in turn activates a cascade of signaling molecules that include small G proteins (see next section for more details) [26]. Finally, in endothelial cells, the VEGFR2 receptor is considered the predominant receptor tyrosine kinase (RTK) which mediates VEGF signaling and induces angiogenesis [42]. The other VEGF receptors are less studied. Nevertheless, researchers have shown that VEGFR1 is significantly overexpressed in distant metastatic ovarian cancer which suggests an important role for the VEGFA/VEGFR1 signaling in the tumor's ability to spread [43]. This was in line with results reported by Dang et al. which showed that increased VEGFR1 expression is associated with poorer levels of progression-free survival and overall lower survival in patients with cervical cancer [44]. Mechanistically, one study demonstrated that VEGF-B/VEGFR1 signaling stimulates the MAPK/ERK pathway in pancreatic carcinoma cell lines [45]. Similarly, VEGFR3 overexpression and activation in different cancers support its role in cancer pathogenesis. VEGFR3 overexpression for example has been observed in the vascular endothelial cells of breast cancer-associated blood vessels [46]. In addition, VEGF-C/VEGFR3 signaling pathway overexpression and overactivation were also reported in colorectal cancer where it was thought to facilitate tumor growth as well as confer the cells the ability to escape the immune system [47]. Alternatively, research has provided significant evidence showing that tumor cells can be induced by VEGF signals in paracrine and autocrine manners that were independent of the VEGFRs. This inferred the presence of other classes of receptors involved in VEGF signaling and consequently led to the discovery of the NRPs. Like the VEGFR, NRPs are now recognized VEGF receptors that contribute to tumor initiation and progression [42, 48–50].

NRP1 and NRP2 are VEGFR receptors which are expressed in vertebrates. Both transmembrane glycoproteins share 44% similarity in terms of their amino acid composition. Unlike VEGFRs, these receptors are comprised of four distinct extracellular domains that are involved in ligand binding as well as a short cytoplasmic domain that does not exhibit any known catalytic activity [51–54]. Several secreted and soluble isoforms of the NRPs as well as NRP2 variants that display differences in their cytoplasmic domains are also generated by alternative splicing [54]. Initially, as the name suggests, NRPs were recognized for the functions they exert during the development of the nervous system where they act as receptors for factors involved in axonal guidance (the semaphorins) [55, 56]. NRPs lack the innate capacity for signaling and therefore can only act as coreceptors. Contingently, in neuronal development, NRPs associate with plexins in order to function as semaphoring receptors [57, 58]. Plexins then induce neuronal development by regulation of the guanosine triphosphates (GTPases). VEGF receptor NRPs are expressed on the surface of many tumor cells [50]. Mechanistically, NRPs form complexes with VEGFR1 and VEGFR2 thereby enhancing the receptor's affinity to VEGF and modulating downstream pathways which are of significant importance for tumor survival and progression [59].

## 5. Rho GTPases

Rho GTPases are small monomeric GTP-binding proteins that were discovered in 1981. The Rho family of GTPases comprises 20 homologues of Rho whose molecular masses range between 20 and 40 kDa [15, 60–62].

These molecules are conserved in plants, mammals, and yeast and belong to the Ras super family whose members share a homology domain [15, 22, 63]. The most studied Rho GTPases are RhoA, RhoC, RhoG, Cdc42, and Rac1. This family regulates different processes including cell growth, differentiation, apoptosis, cell cycle, and gene transcription as well as cell migration and the actin cytoskeleton [15, 60, 62]. Rho GTPases alternate between an active state in which they bind GTP and an inactive state in which they bind GDP [15, 63, 64]. This process is mediated by GTPase-activating proteins (GAPs) which stimulate the innate GTPase activity of GTPases and guanine exchange factors (GEFs) which are phosphatidylinositide 3-kinase- (PI3K-) dependent kinases responsible for the transfer of GTP [15, 63, 65]. Rho GTPases regulate cell motility by modulating the actin cytoskeleton and thus play a critical role in cancer cell migration, invasion, and metastasis [62, 66]. Cdc42, for instance, maintains cell polarity and modulates the actin cytoskeleton in a way which will determine cell movement direction during chemotaxis [67]. Cdc42 can also play also a role in the Rac-dependent formation of lamellipodia, which is initiated by Rac activation of the Arp2/3 complex [17–19, 68]. After the formation of the lamellipodia, Rac stabilizes the newly formed extensions by modulating the extracellular matrix, specifically, the focal complexes [69]. The contractility of the focal complexes is indispensable for cell motility and is mediated by RhoA which also enables the maturation of these complexes [70].

The activation of RhoA has been associated with cancer cell proliferation, progression, and metastasis via the RhoA-Rho-associated protein kinase (ROCK) signaling pathway [71, 72]. This activation is triggered by the binding of cancer cells to the extracellular matrix (ECM) which results in a RhoA-dependent actin recruitment and the formation of nascent adhesions and eventually focal complexes [73–75].

Rac1 upregulation is required for strengthening the adhesion of cancer cells to the ECM [76]. Rac1 suppression however, along with RhoA increased expression levels, also known as the switch between Rac1 and RhoA activation then leads to the separation of the cells from the ECM. This switch thus stimulates cell migration and contributes to cancer progression [77, 78]. RhoA further increases cell invasion by upregulating MMP expression levels [79].

Our lab has further demonstrated that targeting the MAP kinase pathway in astrocytoma cells by using a recombinant anthrax lethal toxin inhibits cell motility and invasion by deregulating the Rho GTPases. In this context, cells treated with the toxin revealed a higher density of stress fibers, potentially indicating an increase in the activity of RhoA [80]. Upon inspection, treated cells did in fact exhibit a higher expression of activated RhoA as well as a higher tendency to adhere properly [80].

RhoB, known for its tumor suppressor role and apoptosis triggering ability in various cancer cell types, can also regulate cell adhesion and migration by modulating the expression levels of the cell surface integrin *β*1 protein [81–83]. Given their important role in cell migration and adhesion, both RhoA and RhoB expression levels were found to decrease as the cells become more malignant [84]. The correlation between the reduction in the levels of integrins and the increased cell migration further highlighted the importance of RhoB regulation of integrin trafficking as a mechanism for controlling cell migration [85].

RhoG also plays a major role in maintaining cell polarity and regulating invasion and migration. This molecule can be activated by the epithelial growth factor (EGF) and hepatocyte growth factor (HGF) and contributes to the formation of lamellipodia and invadopodia [86]. A study has further shown that the RhoG/Rac1 signaling pathway is required for increasing the invasion and migration of salivary adenoid cystic carcinoma [87].

Cdc42 regulation of cell migration and invasion can be very similar to that of Rac1. Indeed, both proteins are activated in the same manner by binding to common GEFs. Upregulated Cdc42 activation and expression levels have thus been linked to increased cancer cell motility and invasiveness and an overall lower survival rate [88].

## 6. Contribution of Rho GTPases in VEGF Signaling

Angiogenesis can occur in a Rho GTPase-dependent manner when VEGF expression levels are modulated by the different members of the Rho family of GTPases. This was observed for example in hepatocellular carcinoma cells where RhoC knockdown reduced the expression of levels of VEGF as well as decreased angiogenesis [13].

Similarly, our lab has demonstrated that the knocking down of RhoC and RhoA in astrocytoma cells decreases the expression levels of VEGF by approximately 25% and 40%, respectively [89]. This highlights the dependence of VEGF expression on both RhoA and RhoC expression levels. After performing tube formation assays and western blots, we further determined that the aforementioned knockdowns prevented angiogenesis. Finally, we proved that RhoA and RhoC induced angiogenesis by increasing VEGF expression [89].

**Figure 1:**
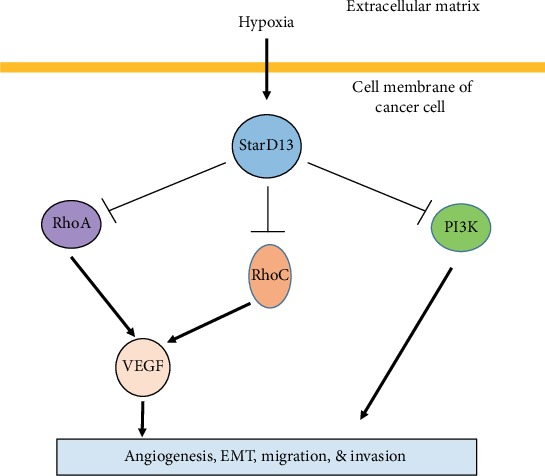
VEGF expression is regulated by Rho GTPases in cancer cells. RhoA, RhoC, and PI3K are involved in VEGF expression in cancer cells leading to angiogenesis and migration. Downregulation of VEGF during hypoxia is due to the overexpression of StarD13 (Rho GAP) which inhibits the activity of Rho A, RhoC, and PI3K.

This was further validated by another study performed in our lab as well. Using pharmacological inhibitors against ERK and PI3K as well as knocking down RhoA and RhoC, we found that ERK and the PI3K/RhoA and RhoC pathway cooperation is required for increasing VEGF expression levels, downstream from EGF. Hypoxia also led to a surprising decrease in the activation of PI3K and RhoA and RhoC. Finally, we showed that the decrease in the activation of RhoA and RhoC GTPases is mediated by a hypoxia-driven overexpression of the StarD13 Rho GAP (Figure 1) [90].

Zhao et al. proved that the upregulation of RhoC in esophageal squamous cell carcinoma could potentially increase the expression of VEGF and hence stimulate tumor invasion and metastasis [91]. The unusual expression of RhoC in ovarian cancer cells further regulated the epithelial-to-mesenchymal transition (EMT) mediated by VEGF and TGF*β*1 signaling [92]. As expected, RhoC overexpression was thus correlated with an increase in metastasis and invasion in bladder cancer [93]. Collectively, these studies and many more demonstrated how the abnormal RhoC expression in multiple cancer cell types contributes to the invasiveness and metastatic ability of cancer cells through VEGF signaling.

**Figure 2:**
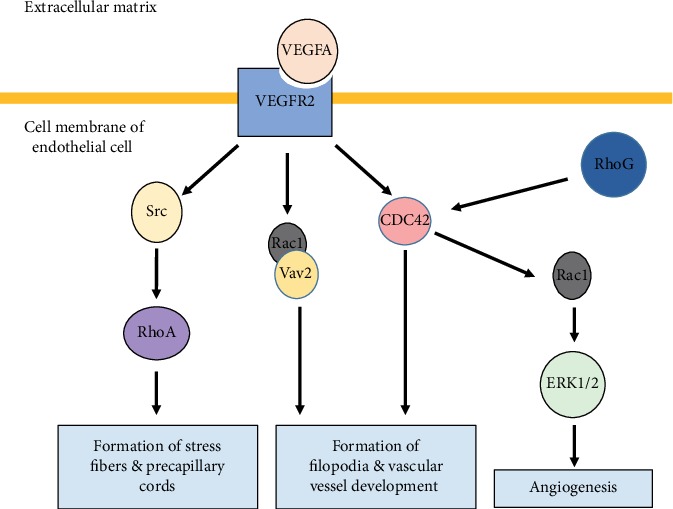
VEGF stimulates angiogenesis in endothelial cells in a Rho GTPase-dependent manner. The VEGFA/VEGF2 axis stimulates the activation of RhoA, Cdc42, and Rac1 leading to vascular development and formation of cytoplasmic migratory structures. Our lab has further proven that RhoG is a positive regulator of angiogenesis in vascular endothelial cells.

In addition, our lab also provided evidence for a direct modulation of tube formation by Rho GTPases. Specifically, our data revealed that RhoG and Rac1 positively regulate tube formation. Mechanistically, this involved RhoG activation of Rac1 and involved Cdc42 activation, which in turn led to increased tube formation in an ERK-dependent manner (Figure 2) [89].

**Figure 3:**
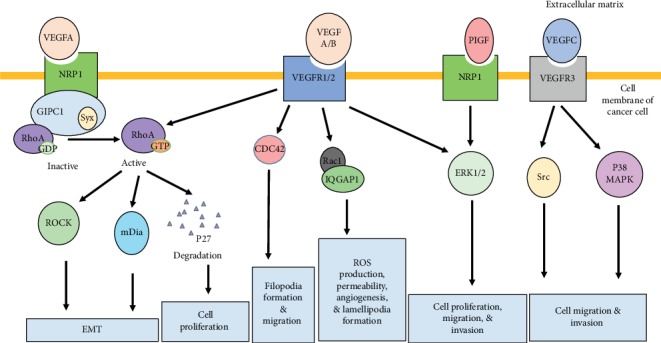
Regulation of cancer hallmarks by the crosstalk between VEGF and Rho GTPase signaling pathways. The formation of cytoplasmic structures, cell migration, invasion, EMT, ROS production, and cell proliferation can be modulated by binding of VEGFA, VEGFB, or VEGFC to their corresponding VEGFR or NRP receptors. VEGFC interacts with Rho GTPases to upregulate Src and p38 MAP kinase activity. VEGFA and VEGFB modulation of cancer hallmarks involves the activation of Rho GTPases and their downstream effectors (ROCK, mDia, and p27) as well as the MAPK pathway.

Other groups have also demonstrated different GTPase-angiogenesis crosstalk mechanisms in cancer cells. For instance, one study revealed that the VEGFA-VEGFR2 axis regulates RhoA, Rac1, and Cdc42 activation [26]. Rac1 also stimulated vascular permeability and cell migration induced by VEGFA [94]. In addition, Rac1 was also involved in the control of the generation of ROS (reactive oxygen species) in a VEGFA-dependent manner [25]. Indeed, phosphorylation by VEGFA resulted in Rac1 forming a complex with the Ras GTPase-activating-like protein 1 (IQGAP1) (Figure 3). Then, the complex stimulated the production of ROS. Rac1 is stabilized by being in the complex, which in turn increased the concentration of Rac1 GTP-bound form [95]. Altogether, this emphasized Rac1's role as a central molecule, which regulates endothelial cell proliferation, migration, permeability, angiogenesis, and lamellipodia formation at the leading edge of cells, which remains the most important role played by a Rho GTPase in migration [96].

With the same mechanisms described above, VEGFR2-stimulated Rac1, RhoA, and Cdc42 activation occurs in endothelial cells. Briefly, VEGFR2 activated Rac1 by inducing the formation of a complex between Rac1 and its GEF Vav2 through the phosphorylation of Vav2 by the protooncogene tyrosine-protein kinase (Src) [97]. Cdc42 activation was mediated by phosphorylation of Tyr1214 on VEGFR2 [97]. This pathway enables actin remodeling specifically in the formation of filopodia as well as the development of vascular vessels (Figure 2) [97].

Downstream of VEGFA and VEGFR-2, RhoA also contributes to VEGF-induced hyperpermeability in the endothelium [98]. VEGFR2 activates Src, which in turn induces the activation of RhoA to cause stress fiber formation [99].

Other effects mediated by RhoA downstream of VEGF include endothelial cell assembly into precapillary cords, as well as increased actomyosin contractility and endothelial cell migration [100].

## 7. Interactions between VEGF and Rho-Related Pathways in Cancer Cells

Cancer metastasis depends on the tumor cell's ability to migrate and invade their surrounding niche. Cells can migrate in an amoeboid or mesenchymal manner. Rho GTPases play an important role in determining how tumor cells will migrate. Specifically, RhoA will favor amoeboid migration, while mesenchymal migration will depend on the action of Rac (Sanz-Moreno, [101]). Cdc42 can contribute to both amoeboid or mesenchymal cell migration, depending on the pathway activating it (Gadea, [102]). As previously described, VEGFA enhances angiogenesis and permeability in newly formed vasculature, and VEGFA activation remains highly regulated by Rho GTPases. Several disorders including the diabetic retinopathy and the macular degeneration are characterized by hyperpermeability and upregulated angiogenesis. In line with these observations, both VEGFA and Rho-related signal inhibitors have been therapeutically explored to counter disease progression [103, 104]. VEGFA and Rho signaling deregulation also promotes cancer progression and metastasis [105–110]. Many researchers have thus shown interest in the therapeutic potential of targeting Rho GTPase signaling and VEGF crosstalk, especially the Rho GTPase signaling and VEGFA crosstalk. However, other VEGFs are tightly regulated by the Rho-related signaling pathways and can thus constitute valuable targets (Figure 3). Both VEGFB and VEGFA stimulate ERK and c-Jun N-terminal kinase (JNK) translocation of p65 to the nucleus, thus promoting colorectal carcinoma cell invasion and metastasis as well as EMT [106] [111]. Similarly, VEGFC binding to its VEGFR3 receptor has also promoted cell motility and cancer invasiveness in different cancer models [112]. VEGFC interaction with Rho GTPases led to the upregulation of Src and p38 MAP kinase activity. This was also influenced by VEGFA [113, 114]. Altogether, this confirms the role of the VEGF-Rho GTPase crosstalk in promoting cancer cell migration and invasiveness. Furthermore, Rho-related signals are also influenced by NRPs and can implicate the activation of VEGFA [115, 116]. This was observed in renal and breast cancer cells, where the overexpression of NRP1 enhanced the Ras/ERK signaling pathway [117] as well as in pancreatic cells which consequently became resistant to chemotherapy [118]. Additionally, studies have demonstrated ERK1/2 phosphorylation in response to the interactions between PIGF and NRP1 and result in tumor growth and spread [119]. This further confirmed the involvement of NRPs in tumorigenesis and tumor progression which are regulated by Rho-related signaling pathways. Similarly, the absence of the expression of VEGFR1 and VEGFR2 in skin and prostate cancer cells as well glioblastoma supports the model according to which VEGFA binding to NRP1 induces Rho signal activation [120]. Researchers have also elucidated that binding of VEGFA to NRP1 leads to the receptor interaction with GIPC1, a scaffold protein and subsequent formation of a complex with the RhoA GEF Syx (Figure 3). This interaction consequently increases the expression levels of active GTP-bound forms of RhoA. Then, activated RhoA degrades p27^kip1^ and promotes cell proliferation. It is worth mentioning that GIPC1 antiapoptotic effects have been observed in breast and colorectal cancer cells [121]. Furthermore, the interaction between GIPC1 and MyoGEF, which is also a RhoA GEF, has been implicated in the activation of RhoA as well as breast cancer invasion [122]. Finally, the effectors of RhoA, ROCK, and mDia1 promote EMT by modulating actin polymerization (Figure 3). This results in tumor invasion through the dissociation of cell junctions [123, 124]. Altogether, this data suggests that the interactions between VEGFA and RhoA pathways could serve as promising targets for novel therapies.

## 8. Conclusion

The Rho GTPases are among the most important molecules for signal transduction in cancer cells. They regulate cytoskeleton remodeling, proliferation, and migration among others. VEGF controls angiogenesis and vascular permeability of endothelial cells and acts downstream of Rho GTPase-related signaling. Our lab has emphasized the roles of Rho GTPases, namely, RhoA, RhoC, and RhoG, in the regulation of angiogenesis by modulating the expression of VEGF or regulating tube formation [89, 90] Other groups have also described a mechanism whereby VEGF receptor interaction with VEGF and VEGF and GEF crosstalk regulate the expression level as well as activity of Rac1 and activate Cdc42. Altogether, this controls the formation of filopodia as well as that of the vascular vessels [97]. Moreover, we described in detail how VEGF also activates RhoA, Rac1, and Cdc42 contributing thus to tumor motility and invasion. Finally, we noticed the complexity and interconnectedness between VEGF and Rho-related pathways which benefit cancer cells in multiple aspects which include but are not limited to angiogenesis, migration, and invasion. Due to the complications associated with the untargeted cancer therapies (chemo- and radiotherapy), approaches targeting specific characteristics and pathways of tumors must be explored [80, 125–129]. Therefore, targeting these interaction factors that are produced and secreted by the tumor cells might exhibit promising therapeutic potential.

## References

[1] Chung A. S., Ferrara N. (2011). Developmental and pathological angiogenesis. *Annual Review of Cell and Developmental Biology*.

[2] Simons M., Gordon E., Claesson-Welsh L. (2016). Mechanisms and regulation of endothelial VEGF receptor signalling. *Nature Reviews Molecular Cell Biology*.

[3] Carmeliet P., Jain R. K. (2000). Angiogenesis in cancer and other diseases. *Nature*.

[4] Zhao L., Xu G., Zhou J. (2006). The effect of RhoA on human umbilical vein endothelial cell migration and angiogenesis in vitro. *Oncology Reports*.

[5] Folkman J. (1995). Angiogenesis in cancer, vascular, rheumatoid and other disease. *Nature Medicine*.

[6] Sellami N., Lamine L. B., Turki A. (2018). Association of VEGFA variants with altered VEGF secretion and type 2 diabetes: a case-control study. *Cytokine*.

[7] Gerber H. P., Dixit V., Ferrara N. (1998). Vascular endothelial growth factor induces expression of the antiapoptotic proteins Bcl-2 and A1 in vascular endothelial cells. *The Journal of Biological Chemistry*.

[8] Gerber H. P., McMurtrey A., Kowalski J. (1998). Vascular endothelial growth factor regulates endothelial cell survival through the phosphatidylinositol 3'-kinase/Akt signal transduction pathway. Requirement for Flk-1/KDR activation. *The Journal of Biological Chemistry*.

[9] Nieves B. J., D'Amore P. A., Bryan B. A. (2009). The function of vascular endothelial growth factor. *BioFactors*.

[10] Nishida N., Yano H., Nishida T., Kamura T., Kojiro M. (2006). Angiogenesis in cancer. *Vascular Health and Risk Management*.

[11] Bryan B. A., D'Amore P. A. (2007). What tangled webs they weave: Rho-GTPase control of angiogenesis. *Cellular and Molecular Life Sciences*.

[12] Bryan B. A., Dennstedt E., Mitchell D. C. (2010). RhoA/ROCK signaling is essential for multiple aspects of VEGF-mediated angiogenesis. *The FASEB Journal*.

[13] Wang W., Wu F., Fang F., Tao Y., Yang L. (2008). RhoC is essential for angiogenesis induced by hepatocellular carcinoma cells via regulation of endothelial cell organization. *Cancer Science*.

[14] Jaffe A. B., Hall A. (2005). Rho GTPases: biochemistry and biology. *Annual Review of Cell and Developmental Biology*.

[15] Bishop A. L., Hall A. (2000). Rho GTPases and their effector proteins. *Biochemical Journal*.

[16] Bravo-Cordero J. J., Sharma V. P., Roh-Johnson M. (2013). Spatial regulation of RhoC activity defines protrusion formation in migrating cells. *Journal of Cell Science*.

[17] Nobes C. D., Hall A. (1995). Rho, Rac and cdc 42 GTPases: regulators of actin structures, cell adhesion and motility. *Biochemical Society Transactions*.

[18] Nobes C. D., Hall A. (1995). Rho, rac, and cdc 42 GTPases regulate the assembly of multimolecular focal complexes associated with actin stress fibers, lamellipodia, and filopodia. *Cell*.

[19] Hall A. (1998). Rho GTPases and the actin cytoskeleton. *Science*.

[20] Hall A., Nobes C. D. (2000). Rho GTPases: molecular switches that control the organization and dynamics of the actin cytoskeleton. *Philosophical Transactions of the Royal Society of London. Series B, Biological Sciences*.

[21] Hotchin N. A., Hall A. (1996). Regulation of the actin cytoskeleton, integrins and cell growth by the Rho family of small GTPases. *Cancer Surveys*.

[22] Fryer B. H., Field J. (2005). Rho, Rac, Pak and angiogenesis: old roles and newly identified responsibilities in endothelial cells. *Cancer Letters*.

[23] Holinstat M., Knezevic N., Broman M., Samarel A. M., Malik A. B., Mehta D. (2006). Suppression of RhoA activity by focal adhesion kinase-induced activation of p190RhoGAP: role in regulation of endothelial permeability. *The Journal of Biological Chemistry*.

[24] Mehta D., Malik A. B. (2006). Signaling mechanisms regulating endothelial permeability. *Physiological Reviews*.

[25] Monaghan-Benson E., Burridge K. (2009). The regulation of vascular endothelial growth factor-induced microvascular permeability requires Rac and reactive oxygen species. *The Journal of Biological Chemistry*.

[26] Vader P., van der Meel R., Symons M. H. (2011). Examining the role of Rac 1 in tumor angiogenesis and growth: a clinically relevant RNAi-mediated approach. *Angiogenesis*.

[27] van der Meel R., Symons M. H., Kudernatsch R. (2011). The VEGF/Rho GTPase signalling pathway: A promising target for anti- angiogenic/anti-invasion therapy. *Drug Discovery Today*.

[28] Bisht M., Dhasmana D. C., Bist S. S. (2010). Angiogenesis: future of pharmacological modulation. *Indian Journal of Pharmacology*.

[29] Maeshima Y., Srivastava R. (2007). Angiogenesis and cancer. *Apoptosis, Cell Signaling, and Human Diseases*.

[30] Jain R. K., di Tomaso E., Duda D. G., Loeffler J. S., Sorensen A. G., Batchelor T. T. (2007). Angiogenesis in brain tumours. *Nature Reviews Neuroscience*.

[31] Ausprunk D. H., Folkman J. (1977). Migration and proliferation of endothelial cells in preformed and newly formed blood vessels during tumor angiogenesis. *Microvascular Research*.

[32] Leung D. W., Cachianes G., Kuang W. J., Goeddel D. V., Ferrara N. (1989). Vascular endothelial growth factor is a secreted angiogenic mitogen. *Science*.

[33] Senger D. R., Galli S. J., Dvorak A. M., Perruzzi C. A., Harvey V. S., Dvorak H. F. (1983). Tumor cells secrete a vascular permeability factor that promotes accumulation of ascites fluid. *Science*.

[34] Koch S., Claesson-Welsh L. (2012). Signal transduction by vascular endothelial growth factor receptors. *Cold Spring Harbor Perspectives in Medicine*.

[35] Claesson-Welsh L., Welsh M. (2013). VEGFA and tumour angiogenesis. *Journal of Internal Medicine*.

[36] Hanahan D., Folkman J. (1996). Patterns and emerging mechanisms of the angiogenic switch during tumorigenesis. *Cell*.

[37] Kerbel R., Folkman J. (2002). Clinical translation of angiogenesis inhibitors. *Nature Reviews. Cancer*.

[38] Wu H., Zhang X., Han D., Cao J., Tian J. (2020). Tumour-associated macrophages mediate the invasion and metastasis of bladder cancer cells through CXCL8. *PeerJ*.

[39] Hansen W., Hutzler M., Abel S. (2012). Neuropilin 1 deficiency on CD4^+^Foxp3^+^ regulatory T cells impairs mouse melanoma growth. *The Journal of Experimental Medicine*.

[40] Shimizu A., Zankov D., Kurokawa-Seo M., Ogita H. (2018). Vascular endothelial growth factor-a exerts diverse cellular effects via small G proteins, Rho and Rap. *International Journal of Molecular Sciences*.

[41] Shibuya M. (2013). Vascular endothelial growth factor and its receptor system: physiological functions in angiogenesis and pathological roles in various diseases. *Journal of Biochemistry*.

[42] Heasman S. J., Ridley A. J. (2008). Mammalian Rho GTPases: new insights into their functions from in vivo studies. *Nature Reviews Molecular Cell Biology*.

[43] Sopo M., Anttila M., Hämäläinen K. (2019). Expression profiles of VEGF-A, VEGF-D and VEGFR1 are higher in distant metastases than in matched primary high grade epithelial ovarian cancer. *BMC Cancer*.

[44] Dang Y. Z., Zhang Y., Li J. P. (2017). High VEGFR1/2 expression levels are predictors of poor survival in patients with cervical cancer. *Medicine (Baltimore)*.

[45] Wey J. S., Fan F., Gray M. J. (2005). Vascular endothelial growth factor receptor-1 promotes migration and invasion in pancreatic carcinoma cell lines. *Cancer*.

[46] Valtola R., Salven P., Heikkilä P. (1999). VEGFR-3 and its ligand VEGF-C are associated with angiogenesis in breast cancer. *The American Journal of Pathology*.

[47] Tacconi C., Ungaro F., Correale C. (2019). Activation of the VEGFC/VEGFR3 pathway induces tumor immune escape in colorectal cancer. *Cancer Research*.

[48] Elaimy A. L., Mercurio A. M. (2018). Convergence of VEGF and YAP/TAZ signaling: implications for angiogenesis and cancer biology. *Science Signaling*.

[49] Soker S., Kaefer M., Johnson M., Klagsbrun M., Atala A., Freeman M. R. (2001). Vascular endothelial growth factor-mediated autocrine stimulation of prostate tumor cells coincides with progression to a malignant phenotype. *The American Journal of Pathology*.

[50] Soker S., Takashima S., Miao H. Q., Neufeld G., Klagsbrun M. (1998). Neuropilin-1 is expressed by endothelial and tumor cells as an isoform-specific receptor for vascular endothelial growth factor. *Cell*.

[51] Geretti E., Shimizu A., Klagsbrun M. (2008). Neuropilin structure governs VEGF and semaphorin binding and regulates angiogenesis. *Angiogenesis*.

[52] Guo H. F., Vander Kooi C. W. (2015). Neuropilin functions as an essential cell surface receptor. *The Journal of Biological Chemistry*.

[53] Parker M. W., Xu P., Li X., Vander Kooi C. W. (2012). Structural basis for selective vascular endothelial growth factor-A (VEGF-A) binding to neuropilin-1. *The Journal of Biological Chemistry*.

[54] Rossignol M., Gagnon M. L., Klagsbrun M. (2000). Genomic organization of human neuropilin-1 and neuropilin-2 genes: identification and distribution of splice variants and soluble isoforms. *Genomics*.

[55] Chen H., Chédotal A., He Z., Goodman C. S., Tessier-Lavigne M. (1997). Neuropilin-2, a novel member of the neuropilin family, is a high affinity receptor for the semaphorins Sema E and Sema IV but not Sema III. *Neuron*.

[56] Kolodkin A. L., Levengood D. V., Rowe E. G., Tai Y. T., Giger R. J., Ginty D. D. (1997). Neuropilin is a semaphorin III receptor. *Cell*.

[57] Takahashi T., Fournier A., Nakamura F. (1999). Plexin-neuropilin-1 complexes form functional semaphorin-3A receptors. *Cell*.

[58] Winberg M. L., Noordermeer J. N., Tamagnone L. (1998). Plexin A is a neuronal semaphorin receptor that controls axon guidance. *Cell*.

[59] Neufeld G., Kessler O., Herzog Y. (2002). The interaction of neuropilin-1 and neuropilin-2 with tyrosine-kinase receptors for VEGF. *Advances in Experimental Medicine and Biology*.

[60] Al-Koussa H., El Atat O., Jaafar L., Tashjian H., El-Sibai M. (2020). The role of Rho GTPases in motility and invasion of glioblastoma cells. *Analytical Cellular Pathology (Amsterdam)*.

[61] Burridge K., Wennerberg K. (2004). Rho and Rac take center stage. *Cell*.

[62] Ridley A. J. (2015). Rho GTPase signalling in cell migration. *Current Opinion in Cell Biology*.

[63] Hanna S., El-Sibai M. (2013). Signaling networks of Rho GTPases in cell motility. *Cellular Signalling*.

[64] Boettner B., Van Aelst L. (2002). The role of Rho GTPases in disease development. *Gene*.

[65] Schmidt A., Hall A. (2002). Guanine nucleotide exchange factors for Rho GTPases: turning on the switch. *Genes & Development*.

[66] Lauffenburger D. A., Horwitz A. F. (1996). Cell migration: a physically integrated molecular process. *Cell*.

[67] El-Sibai M., Nalbant P., Pang H. (2007). Cdc 42 is required for EGF-stimulated protrusion and motility in MTLn3 carcinoma cells. *Journal of Cell Science*.

[68] Condeelis J. S., Wyckoff J. B., Bailly M. (2001). Lamellipodia in invasion. *Seminars in Cancer Biology*.

[69] Rottner K., Hall A., Small J. V. (1999). Interplay between Rac and Rho in the control of substrate contact dynamics. *Current Biology*.

[70] Kaverina I., Krylyshkina O., Small J. V. (2002). Regulation of substrate adhesion dynamics during cell motility. *The International Journal of Biochemistry & Cell Biology*.

[71] Garcia-Cattaneo A., Braga V. M. M. (2013). Hold on tightly: how to keep the local activation of small GTPases. *Cell Adhesion & Migration*.

[72] Trepat X., Chen Z., Jacobson K. (2012). Cell migration. *Comprehensive Physiology*.

[73] Alexandrova A. Y., Arnold K., Schaub S. (2008). Comparative dynamics of retrograde actin flow and focal adhesions: formation of nascent adhesions triggers transition from fast to slow flow. *PLoS One*.

[74] Goldberg L., Kloog Y. (2006). A Ras inhibitor tilts the balance between Rac and Rho and blocks phosphatidylinositol 3-kinase-dependent glioblastoma cell migration. *Cancer Research*.

[75] Parsons J. T., Horwitz A. R., Schwartz M. A. (2010). Cell adhesion: integrating cytoskeletal dynamics and cellular tension. *Nature Reviews Molecular Cell Biology*.

[76] Manning T. J., Parker J. C., Sontheimer H. (2000). Role of lysophosphatidic acid and rho in glioma cell motility. *Cell Motility and the Cytoskeleton*.

[77] Hernández S. E., Settleman J., Koleske A. J. (2004). Adhesion-dependent regulation of p190RhoGAP in the developing brain by the Abl-related gene tyrosine kinase. *Current Biology*.

[78] Ren X. D., Kiosses W. B., Schwartz M. A. (1999). Regulation of the small GTP-binding protein Rho by cell adhesion and the cytoskeleton. *The EMBO Journal*.

[79] Abécassis I., Olofsson B., Schmid M., Zalcman G., Karniguian A. (2003). RhoA induces MMP-9 expression at CD44 lamellipodial focal complexes and promotes HMEC-1 cell invasion. *Experimental Cell Research*.

[80] Al-Dimassi S., Salloum G., Saykali B. (2016). Targeting the MAP kinase pathway in astrocytoma cells using a recombinant anthrax lethal toxin as a way to inhibit cell motility and invasion. *International Journal of Oncology*.

[81] Croft D. R., Olson M. F. (2011). Transcriptional regulation of Rho GTPase signaling. *Transcription*.

[82] Prendergast G. C. (2001). Farnesyltransferase inhibitors define a role for RhoB in controlling neoplastic pathophysiology. *Histology and Histopathology*.

[83] Wheeler A. P., Ridley A. J. (2007). RhoB affects macrophage adhesion, integrin expression and migration. *Experimental Cell Research*.

[84] Forget M. A., Desrosiers R. R., Del M. (2002). The expression of rho proteins decreases with human brain tumor progression: potential tumor markers. *Clinical & Experimental Metastasis*.

[85] Vega F. M., Colomba A., Reymond N., Thomas M., Ridley A. J. (2012). RhoB regulates cell migration through altered focal adhesion dynamics. *Open Biology*.

[86] Kwiatkowska A., Didier S., Fortin S. (2012). The small GTPase RhoG mediates glioblastoma cell invasion. *Molecular Cancer*.

[87] Xu Z. D., Hao T., Gan Y. H. (2020). RhoG/Rac1 signaling pathway involved in migration and invasion of salivary adenoid cystic carcinoma cells. *Oral Diseases*.

[88] Okura H., Golbourn B. J., Shahzad U. (2016). A role for activated Cdc42 in glioblastoma multiforme invasion. *Oncotarget*.

[89] El Atat O., Fakih A., El-Sibai M. (2019). RHOG activates RAC1 through CDC42 leading to tube formation in vascular endothelial cells. *Cells*.

[90] Nicolas S., Abdellatef S., Haddad M. A., Fakhoury I., el-Sibai M. (2019). Hypoxia and EGF stimulation regulate VEGF expression in human glioblastoma multiforme (GBM) cells by differential regulation of the PI3K/Rho-GTPase and MAPK pathways. *Cells*.

[91] Zhao Z. H., Tian Y., Yang J. P., Zhou J., Chen K. S. (2015). RhoC, vascular endothelial growth factor and microvascular density in esophageal squamous cell carcinoma. *World Journal of Gastroenterology*.

[92] Gou W.-F., Zhao Y., Lu H. (2014). The role of RhoC in epithelial-to-mesenchymal transition of ovarian carcinoma cells. *BMC Cancer*.

[93] Kamai T., Tsujii T., Arai K. (2003). Significant association of Rho/ROCK pathway with invasion and metastasis of bladder cancer. *Clinical Cancer Research*.

[94] Eriksson A., Cao R., Roy J. (2003). Small GTP-binding protein Rac is an essential mediator of vascular endothelial growth factor-induced endothelial fenestrations and vascular permeability. *Circulation*.

[95] Yamaoka-Tojo M., Ushio-Fukai M., Hilenski L. (2004). IQGAP1, a novel vascular endothelial growth factor receptor binding protein, is involved in reactive oxygen species--dependent endothelial migration and proliferation. *Circulation Research*.

[96] Yamazaki D., Suetsugu S., Miki H. (2003). WAVE2 is required for directed cell migration and cardiovascular development. *Nature*.

[97] Lamalice L., Houle F., Jourdan G., Huot J. (2004). Phosphorylation of tyrosine 1214 on VEGFR2 is required for VEGF-induced activation of Cdc42 upstream of SAPK2/p38. *Oncogene*.

[98] Sun H., Breslin J. W., Zhu J., Yuan S. Y., Wu M. H. (2006). Rho and ROCK signaling in VEGF-induced microvascular endothelial hyperpermeability. *Microcirculation*.

[99] Dejana E. (2004). Endothelial cell-cell junctions: happy together. *Nature Reviews Molecular Cell Biology*.

[100] Hoang M. V., Whelan M. C., Senger D. R. (2004). Rho activity critically and selectively regulates endothelial cell organization during angiogenesis. *Proceedings of the National Academy of Sciences of the United States of America*.

[101] Gadea G., Sanz-Moreno V., Self A., Godi A., Marshall C. J. (2008). DOCK10-mediated Cdc42 activation is necessary for amoeboid invasion of melanoma cells. *Current Biology*.

[102] Sanz-Moreno V., Gadea G., Ahn J. (2008). Rac activation and inactivation control plasticity of tumor cell movement. *Cell*.

[103] Bolinger M. T., Antonetti D. A. (2016). Moving past anti-VEGF: novel therapies for treating diabetic retinopathy. *International Journal of Molecular Sciences*.

[104] Yamaguchi M., Nakao S., Arima M. (2017). Rho-kinase/ROCK as a potential drug target for vitreoretinal diseases. *Journal of Ophthalmology*.

[105] Chen S., Wang J., Gou W. F. (2013). The involvement of RhoA and Wnt-5a in the tumorigenesis and progression of ovarian epithelial carcinoma. *International Journal of Molecular Sciences*.

[106] Fan F., Wey J. S., McCarty M. F. (2005). Expression and function of vascular endothelial growth factor receptor-1 on human colorectal cancer cells. *Oncogene*.

[107] Inoue M., Hager J. H., Ferrara N., Gerber H. P., Hanahan D. (2002). VEGF-A has a critical, nonredundant role in angiogenic switching and pancreatic beta cell carcinogenesis. *Cancer Cell*.

[108] Mirones I., Conti C. J., Martínez J., Garcia M., Larcher F. (2009). Complexity of VEGF responses in skin carcinogenesis revealed through ex vivo assays based on a VEGF-A null mouse keratinocyte cell line. *The Journal of Investigative Dermatology*.

[109] Park S. T., Kim B. R., Park S. H. (2014). Suppression of VEGF expression through interruption of the HIF-1*α* and Akt signaling cascade modulates the anti-angiogenic activity of DAPK in ovarian carcinoma cells. *Oncology Reports*.

[110] Xue Y., Bi F., Zhang X. (2006). Role of Rac1 and Cdc42 in hypoxia induced p53 and von Hippel-Lindau suppression and HIF1alpha activation. *International Journal of Cancer*.

[111] Bates R. C., Goldsmith J. D., Bachelder R. E. (2003). Flt-1-dependent survival characterizes the epithelial-mesenchymal transition of colonic organoids. *Current Biology*.

[112] Su J. L., Yang P. C., Shih J. Y. (2006). The VEGF-C/Flt-4 axis promotes invasion and metastasis of cancer cells. *Cancer Cell*.

[113] Aesoy R., Sanchez B. C., Norum J. H., Lewensohn R., Viktorsson K., Linderholm B. (2008). An autocrine VEGF/VEGFR2 and p38 signaling loop confers resistance to 4-hydroxytamoxifen in MCF-7 breast cancer cells. *Molecular Cancer Research*.

[114] Oommen S., Gupta S. K., Vlahakis N. E. (2011). Vascular endothelial growth factor A (VEGF-A) induces endothelial and cancer cell migration through direct binding to integrin {alpha}9{beta}1: identification of a specific {alpha}9{beta}1 binding site. *The Journal of Biological Chemistry*.

[115] Nguyen Q. D., Rodrigues S., Rodrigue C. M. (2006). Inhibition of vascular endothelial growth factor (VEGF)-165 and semaphorin 3A-mediated cellular invasion and tumor growth by the VEGF signaling inhibitor ZD4190 in human colon cancer cells and xenografts. *Molecular Cancer Therapeutics*.

[116] Shimizu A., Mammoto A., Italiano J. E. (2008). ABL2/ARG tyrosine kinase mediates SEMA3F-induced RhoA inactivation and cytoskeleton collapse in human glioma cells. *The Journal of Biological Chemistry*.

[117] Cao Y., E G., Wang E. (2012). VEGF exerts an angiogenesis-independent function in cancer cells to promote their malignant progression. *Cancer Research*.

[118] Wey J. S., Gray M. J., Fan F. (2005). Overexpression of neuropilin-1 promotes constitutive MAPK signalling and chemoresistance in pancreatic cancer cells. *British Journal of Cancer*.

[119] Snuderl M., Batista A., Kirkpatrick N. D. (2013). Targeting placental growth factor/neuropilin 1 pathway inhibits growth and spread of medulloblastoma. *Cell*.

[120] Yoshida A., Shimizu A., Asano H. (2015). VEGF-A/NRP1 stimulates GIPC1 and Syx complex formation to promote RhoA activation and proliferation in skin cancer cells. *Biology Open*.

[121] Chittenden T. W., Pak J., Rubio R. (2010). Therapeutic implications of GIPC1 silencing in cancer. *PLoS One*.

[122] Wu D., Haruta A., Wei Q. (2010). GIPC1 interacts with MyoGEF and promotes MDA-MB-231 breast cancer cell invasion. *The Journal of Biological Chemistry*.

[123] Hernández-García R., Iruela-Arispe M. L., Reyes-Cruz G., Vázquez-Prado J. (2015). Endothelial RhoGEFs: a systematic analysis of their expression profiles in VEGF-stimulated and tumor endothelial cells. *Vascular Pharmacology*.

[124] Lamouille S., Xu J., Derynck R. (2014). Molecular mechanisms of epithelial-mesenchymal transition. *Nature Reviews. Molecular Cell Biology*.

[125] Abou-Antoun T. J., Nazarian J., Ghanem A., Vukmanovic S., Sandler A. D. (2018). Molecular and functional analysis of anchorage independent, treatment-evasive neuroblastoma tumorspheres with enhanced malignant properties: a possible explanation for radio-therapy resistance. *PLoS One*.

[126] Al Hassan M., Fakhoury I., El Masri Z. (2018). Metformin treatment inhibits motility and invasion of glioblastoma cancer cells. *Analytical Cellular Pathology (Amsterdam)*.

[127] El-Amm J., Aragon-Ching J. B. (2016). Targeting bone metastases in metastatic castration-resistant prostate cancer. *Clinical Medicine Insights: Oncology*.

[128] Nader R., El Amm J., Aragon-Ching J. B. (2018). Role of chemotherapy in prostate cancer. *Asian Journal of Andrology*.

[129] Nasreddine G., El-Sibai M., Abi-Habib R. J. (2020). Cytotoxicity of [HuArgI (co)-PEG 5000]-induced arginine deprivation to ovarian cancer cells is autophagy dependent. *Investigational New Drugs*.

